# Factors and Mechanisms for Pharmacokinetic Differences between Pediatric Population and Adults

**DOI:** 10.3390/pharmaceutics3010053

**Published:** 2011-02-07

**Authors:** Eva Fernandez, Raul Perez, Alfredo Hernandez, Pilar Tejada, Marta Arteta, Jose T. Ramos

**Affiliations:** 1 Department of Pharmacy, Getafe University Hospital, Carretera Toledo Km 12,5 Getafe, Madrid, Spain; 2 Department of Paediatrics, Getafe University Hospital, Carretera Toledo Km 12,5 Getafe, Madrid, Spain

**Keywords:** pharmacokinetics, children, neonates, infants, bioavailability, distribution, metabolism, excretion

## Abstract

Many physiologic differences between children and adults may result in age-related changes in pharmacokinetics and pharmacodynamics. Factors such as gastric pH and emptying time, intestinal transit time, immaturity of secretion and activity of bile and pancreatic fluid among other factors determine the oral bioavailability of pediatric and adult populations. Anatomical, physiological and biochemical characteristics in children also affect the bioavailability of other routes of administration.

Key factors explaining differences in drug distribution between the pediatric population and adults are membrane permeability, plasma protein binding and total body water. As far as drug metabolism is concerned, important differences have been found in the pediatric population compared with adults both for phase I and phase II metabolic enzymes. Immaturity of glomerular filtration, renal tubular secretion and tubular reabsorption at birth and their maturation determine the different excretion of drugs in the pediatric population compared to adults.

## 1. Introduction

The statement that children are not small adults is valid particularly in pediatric clinical pharmacology. The application of pharmacokinetic and pharmacodynamic knowledge to the pediatric field implies the understanding of the maturing process in a continuing changeable organism at every age, from preterm neonates to adolescence.

Pharmacokinetics studies the passage of the drugs through the organism, this means liberation, absorption, distribution, metabolism and excretion (LADME). In other words, while pharmacology studies the effect of drugs in the organism, pharmacokinetics study the effects suffered by the drug when in contact with the organism. Pharmacodynamics refers to the relationship between drug dosage and effect in a certain organ or system.

Anatomical, physiological and biochemical changes that occur from birth affect pharmacokinetics/pharmacodynamics and therefore the bioavailability of drugs.

In this article, we review the way pharmacokinetic parameters are affected through the development and maturing process in children, which determine the disposition of drugs and thus the necessity to carry out specific studies in the different pediatric ages and to establish particular dosification steps in the pediatric population.

## 2. Pharmacokinetic Parameters

The concentration of a drug attained after a single dose depends on its volume of distribution, which in turn depends on the volume of plasma and tissue and on the fractions of unbound drug in plasma and tissue. After multiple dosing, mean steadystate concentrations reflect the dose and dosage interval, clearance, and bioavailability. Total clearance is based on the sum of the partial metabolic and renal clearances.

Some pharmacokinetic parameters such as clearance, volume of distribution and bioavailability are age-related. This affects the dose and dosage interval needed to maintain therapeutic concentrations.

Table 1 shows pediatric indication for some drugs by the FDA.

### 2.1. Absorption

Several methods are used to administer drugs to children, but as with adults, the most common involve extravascular routes. A therapeutic agent administered by means of any extravascular route must overcome chemical, physical, mechanical and biological barriers in order to be absorbed.

Developmental changes in absorptive surfaces, especially the gastrointestinal tract, can influence the rate and extent of the bioavailability of a drug. Also, physiopathological factors such as shock cause hypoxia and hypoperfusion and therefore reduce the absorption of drugs.

#### 2.1.1. Oral administration

Oral administration is the eligible route whenever possible.

##### 2.1.1.1. Gastric pH

At birth, pH is practically neutral (6-8), then falls to approximately 1-3 within the first 24 h following birth, and later on gradually returns to neutrality by day 10 [1,2]. It slowly declines again thereafter to reach adult values. By the age of three years, the amount of gastric acid excreted per kilogram of body weight is similar to that excreted in adults, thus reaching the same pH values (2-3) [3].

These initial changes do not occur in premature infants, who seem to have little or no free acid during the first 14 days of life [2]. The gastric acid differences may affect the dissolution and absorption of drugs:

- Acid labile drugs such as ampicillin, erythromycin or amoxycillin are more efficiently absorbed when orally administered in the neonate and infant than in the adult [4,5].
- Weak organic acids such as phenytoin and phenobarbital have a decreased absorption. Thus, bioavailability of the enteral formulation of phenytoin is 75% in neonates and infants up to four months compared with nearly complete absorption in adults [6].
- Basic drugs are absorbed more rapidly than in adults [2].

##### 2.1.1.2. Gastric emptying

In normal adults, gastric emptying is biphasic, a rapid (10-20 min) first phase is followed by an exponentially slower phase [7]. In the preterm infant, gastric emptying is slow and linear. It approaches adult values within the first 6-8 months of life [1,8].

It would be expected that drugs might have an improved absorption rate in young infants, owing to prolonged contact with the gastrointestinal mucosa secondary to slow gastric emptying. However, data suggest that certain drugs, including amoxycillin, rifampin and chloramphenicol, demonstrate delayed and incomplete absorption in neonates and small infants [9-11].

##### 2.1.1.3. Intestinal transit

Few studies have systematically evaluated the effect of developmental changes on drug absorption in infants and children. Intestinal transit time is prolonged in neonates because of reduced motility and peristalsis, but appears to be reduced in older infants as a result of increased intestinal motility and it seems to be responsible, together with other factors, for the incomplete absorption of some sustained release formulations, as has been extensively demonstrated for theophylline [6].

##### 2.1.1.4. Other factors

- Immaturity of secretion and activity of bile and pancreatic fluid leads to impaired fat digestion in neonates and infants in the first few months. The absorption of fat-soluble vitamins (vitamin D and E) is reduced in neonates, probably because of the inadequate bile salt pool in the ileum [4]. This fact needs to be taken into account when administering fat soluble substances to neonates and young children and dose adjustment must be carried out. After a few months, the infant is capable of efficiently absorbing fat-soluble compounds because of a postnatal maturation of bile salt [12,13].
- Immaturity of the intestinal mucosa [2] is characterized by lower intestinal motility and proteolytic enzymatic activity, reduced IgA secretion, diminished number of linfocites B and higher intestinal permeability. The potential consequences of these characteristics are an abnormal bacterial colonization of the superior gastrointestinal tract, an insufficient protein digestion, lower defensive capacity and higher absorption of proteins, immunoglobulins, carbohydrates, bacteria, virus, and toxins [14].
- High levels of intestinal beta-glucuronidase activity [2].
- Immaturity of transport systems [15,16]: gabapentin is absorbed through a L-amino acid transporter in the gastrointestinal mucosa and is excreted by the kidney as unchanged drug. Its absorption process is saturable, therefore its bioavailability is dose dependent. Because renal clearance reaches adult levels at 1-2 years of age, and because gabapentin is not protein bound, the higher oral clearance found is due to decreased bioavailability resulting from immature activity of the L-amino acid transporter, which limits absorption.
- Reduced first-pass metabolism. This topic will be addressed in the metabolism section.
- Variable microbial colonization: during fetal life, the gastrointestinal tract is sterile. From birth, microbial colonization occurs and bacteria are detected within 4-8 hours. The digestive tract colonization influences the bile salts metabolism and gastrointestinal motility. The types of bacteria that colonize the digestive tract of the full-term neonates are different depending on whether the neonate receives maternal or artificial milk [17]. The bioavailability of some drugs is influenced by the metabolism (hydrolysis and reductions) by the intestinal microflora, which is different in infants, children and adults. The capacity to metabolize biliar acids is reached by four years but the metabolism of drugs is unknown. By two years there are bacteria in the intestine able to metabolize digoxin, however, it is not until adolescence when adult levels are reached [17].

#### 2.1.2. Intramuscular administration

The bioavailability of drugs after intramuscular injection depends on the perfusion in the area of the injection, the rate of drug penetration through the capillary endothelium, and the apparent volume into which the drug has been distributed. Several physiological factors distinguish neonates from older children and adults. First of all, a decrease of the blood flow to muscle, which varies quite considerably over the first 2-3 weeks of life, less muscular mass and a higher proportion of water [4,7]. Intramuscular administration of drugs is unreliable in neonates and the pharmacokinetics are unpredictable, although for drugs such as aminoglycosides and ampicillin, the time needed to achieve peak concentration is comparable for infants, children and adults when administered by intramuscular route [18,19].

#### 2.1.3. Rectal administration

Rectal Administration is a useful route if the patient is unable to take drugs by mouth and the intravenous administration is difficult. The rectal area is small but well vascularized, and the absorption occurs through superior, media and inferior hemorrhoidal veins.

The rectal route is not much modified by maturation. The local pH of the rectum is close to neutral in adults, but alkaline in most children [2]. The first pass effect may have some effect on the bioavailability of rectal administrations. Whereas drugs administered low in the rectum are delivered systemically before passing through the liver, drugs administered high in the rectum are usually carried directly to the liver and therefore are subject to metabolism and the enterohepatic circulation [20]. Therefore, depending on the absorption site of the rectum, bioavailability is expected to vary between neonates, infants, children and adults.

However, ketoprofen has a similar absorption in children and adults after rectal administration [21]. A prolonged absorption time of paracetamol was shown in preterm neonates in comparison with term neonates, possibly due to differences in rectal temperature [22]. The bioavailability of paracetamol seems to decrease with age, likely because of an increase in the first-pass effect of the liver by maturation of liver enzymes. Apparently there are differences in the degree of absorption after rectal administration of tramadol between children and adults, probably due to the pH difference [23].

#### 2.1.4. Percutaneous administration

Two major factors determine the rate and extent of percutaneous absorption. The thickness of the epidermal stratum corneum is inversely related to absorption, whereas the state of skin hydration directly influences absorption [7]. This fact, together with a greater body surface area related to weight, may cause excessive absorption of an agent applied to the skin in the neonate and small infants.

Data from *in vivo* studies indicate an inverse correlation between permeability and gestational age. Permeability rates were 100- to 1000-fold greater before 30 weeks gestation and full-term neonates have a 3- to 4-fold greater permeation rate than adults [24].

Hydrogel of theophylline has been used for the treatment of apnoea in the newborn, showing adequate plasma concentrations until three months of life, the absorption is reduced afterwards [25].

However, systemic toxicity can be seen with the percutaneous administration of drugs, such as lidocaine and corticosteroids during the first 8-12 months [26].

#### 2.1.5. Intrapulmonary administration

Intrapulmonary administration of drugs (inhalation) is increasingly being used in infants and children.

Apart from general anesthetics, the principal goal of this route of administration is to achieve a predominantly local effect, however systemic exposure does occur. Developmental changes in the architecture of the lung and its ventilatory capacity alter the absorption after the intrapulmonary administration of a drug [4].

#### 2.1.6. Intranasal administration

There are many advantages of intranasal administration of drugs including ease of administration, speed of action, good tolerance and not having hepatic first-pass effect [27]. However, limited volume of administration and poor absorption of hydrophilic drugs account for disadvantages [28]. Some drugs that have been tested intranasally in children are midazolam, fentanyl, butorphanol, ketamine, sufentanil, corticosteroids, antihistamines, sumatriptan and desmopressin [27-31].

In a randomized double-blind study in pediatric patients with a mean age of 4.5 years who underwent burn healing, intranasal fentanyl (1.4 mcg/kg) was compared with oral morphine concluding equivalence in analgesic efficacy between both drugs, but with a time of less action and fewer adverse effects when administered intranasally [29].

### 2.2. Distribution

After absorption, a drug is distributed to various body compartments according to its physiochemical properties, such as molecular size, ionization constant, and relative aqueous and lipid solubility.

Several of the processes involved in the distribution of drugs are clearly different in neonates and infants when compared to adults.

Factors including plasma protein binding and water partitioning are continuously fluctuating throughout the first years of life, thus affecting the distribution of drugs.

#### 2.2.1. Membrane permeability

At birth, the blood-brain barrier (BBB) is still not fully mature and medicinal products may gain access to the central nervous system with resultant toxicity.

This neonatal greater permeability in turn allows some drugs with low penetration capacity to achieve higher concentrations in brain than those reached in children or adults, as it has been described with amphotericin B [32].

As the brain is disproportionately large in young children, this factor, combined with the immaturity of the BBB, leads to a significant additional volume for chemical partitioning. The volume of the central nervous system (CNS) is relatively large in younger children and does not correlate well with body surface area (BSA) in the pediatric population since CNS volume reaches 80-90% of adult values by age 4-6 years, yet BSA does not reach adult values until about age 16-18 years. This suggests that BSA dosing of intrathecal therapy would yield relatively lower cerebrospinal fluid concentrations (CFC) in younger children *versus* adolescents and adults. For example, CFC following the intrathecal injection of methotrexate (based on BSA and administered to patients ranging 3-39 years old) were found to vary 100-fold, with lower concentrations observed in younger children [33].

#### 2.2.2. Plasma protein binding

Plasma protein binding of compounds is dependent on the amount of available binding proteins, the number of available binding sites, the affinity constant of the drug for the protein(s), and the presence of pathophysiological conditions or endogenous compounds that may alter the drug-protein binding interaction.

In general, acidic drugs mainly bind to albumin, whereas basic drugs bind to globulins, α1-acid glycoprotein (AAG) and lipoproteins [4].

Frequently, the unbound fraction is higher in neonates and infants for several reasons. First, the concentration of binding proteins may be reduced [34]. Moreover, these proteins are qualitatively different and generally have lower binding capacities, especially in neonates [34,35]. Furthermore, physiological and pathological increases in bilirubin and free fatty acid plasma concentrations are often present in the neonatal period. Increased concentrations of nonesterified fatty acids reduce drug binding, as also occurs by the increased levels of bilirubin and other endogenous substances competitively binding to albumin [36].

The concentration of AAG, also low at birth, increases over the first year to reach adult values [37]. The lower concentration of AAG in newborns and infants probably accounts for the decrease in protein binding of sufentanyl in these age groups compared with that in older children or adults (the free fraction of sufentanyl is 20% in newborns compared with 12% in infants and 8% in children and adults) [38].

#### 2.2.3. Body water

In very young infants, the total body water is high (80-90% of the body weight (BW)) while fat content is low (10-15% BW). The amount of total body water decreases to 55-60% by adulthood [33,36]. The extracellular water content is about 45% in neonates, and especially large in neonates with low birth weights, compared with 20% in adulthood [33].

These changes will result in a relatively higher volume of distribution of water-soluble drugs in pediatric population than in adulthood, such as gentamicin (0.5-1.2 l/kg in neonates and infants and 0.2-0.3 l/kg in adults) [6], linezolid [39], phenobarbital [40] or propofol [41], and similar or lower for fat-soluble drugs such as diazepam [2].

### 2.3. Metabolism

The liver is quantitatively by far the most important organ for drug metabolism. It constitutes 5% of the BW at birth but only 2% in adults [42]. The hepatic clearance depends on several factors, including blood flow, hepatic enzyme activities (intrinsic metabolism), transport systems and plasma protein binding. Blood flow and drug-metabolising enzymes are reduced in children; the former reaches adult rates by around one year of age [16].

The primary objective of drug metabolism is to transform drugs into more water soluble substances to facilitate their excretion. This process occurs primarily in liver hepatocytes to generate metabolites that are inactive and relatively non-toxic; however, metabolites may occasionally be the source of toxic effects.

Drug metabolism mechanisms can be classified into phase I, involving structural alteration of the drug molecule, and phase II reactions, consisting of conjugation with another often more water-soluble moiety.

Phase I reactions can be oxidation, reduction and hydrolysis. Oxidative reactions are the most important and frequently, though not necessarily, cytochrome P450 (CYP)-dependent.

At birth, both phase I and II metabolic enzymes may be immature. The different capacity to metabolize drugs in children may result in higher or lower drug plasma levels than those reached in adults [33].

In fact, there are examples of therapeutic agents that produce metabolites in children that are not normally present in adults. These metabolites may be responsible for some of the efficacy and/or toxicity observed with drug administration in children, an example is caffeine production in neonates receiving theophylline [42].

#### 2.3.1. Phase I reactions

##### 2.3.1.1. CYP system

The CYP isoenzyme superfamily comprises over 50 proteins located in the lipophilic membranes of the smooth endoplasmic reticulum of the liver and other tissues in vesicles called microsomes. Total cytochrome P450 content in the fetal liver is between 30 and 60% of adult values and approaches adult values by 10 years of age [43]. The CYP families 1–4 are mainly involved in xenobiotic metabolism, while other CYP families are mainly involved in the metabolism of endogenous substrates [42].

###### CYP1A

CYP1A2 accounts for approximately 13% of the total cytochrome P450 enzyme expression in the liver of healthy adult humans [43].The CYP1A subfamily consists of two isoforms, CYP1A1 and 1A2. The latter is barely detectable in early neonatal microsomes, becomes readily detectable in infants aged one to three months, is present at about 30% of the adult level in infants <1 year, 81% at two years and comparable to adult values in children of three years or more [6]. CYP1A2 is involved in all demethylations (N1, N3, N7) in the metabolism of caffeine. Total demethylation and *N*3- and *N*7-demethylation increase exponentially with postnatal age, whereas the maturation of N1 demethylation is delayed and does not occur until after 19 months of age. *N*3-demethylation is more important in young infants than adults values [44].

Clearance of caffeine is decreased in the neonate reaching the adult rate of elimination by 5-6 months of age [45]. Results of the caffeine breath test to measure caffeine clearance are 50% higher in children aged 3-9 years and 33% higher in children and adolescents aged 9-15 years than in adults [46].

Theophylline is classically characterized as a CYP1A2 substrate with minor metabolism by CYP2E1 and CYP3A4. Clearance of theophylline is approximately 50% of adult levels in neonates. It increases to 50% greater than adult values by five years of age and decreases to adult values by 15 years [47].

Therefore, to achieve equivalent target plasma concentrations, we predict that doses (in milligrams/kilogram) for drugs metabolized by CYP1A2 should be reduced by approximately 50% in neonates. Children aged 2-10 years may require doses approximately 50% higher than adults, and adolescents may need doses similar to those prescribed for adults [16].

###### CYP2C

The isozymes of the CYP2C subfamily are involved in the metabolism of a number of therapeutic agents, such as anticonvulsants, non-steroidal anti-inflammatory drugs as well as omeprazole, warfarin, tolbutamide, diazepam, propranolol and endogenous agents such as arachidonic acid [42]. CYP2C9, 2C19 and 2C8 have been extensively studied and are the most relevant isoenzymes.

The CYP2C isozymes are barely detectable in newborns; they represent one-third of the adult value at one month and remain unchanged until one year [48].

The developmental expression of two isoenzymes of the CYP2C subfamily has been investigated. Liver microsomal CYP2C9 and -2C19 were measured (ages ranging from eight weeks gestation to 18 years). CYP2C9-specific content and catalytic activity were consistent with expression at 1-2% of mature values during the first trimester, with progressive increases during the second and third trimesters to levels >30% of mature values. From birth to five months, CYP2C9 reaches 50% of mature level activity. Less variable CYP2C9 protein and activity values were observed between five months and 18 years [49].

Phenytoin, mainly metabolized by this isoenzyme, displays age-dependent Michaelis-Menten kinetics. Thus, in preterm infants, the apparent half-life of phenytoin is prolonged (75 h) relative to term infants <1 week after birth (20 h) or term infants aged >2 weeks (8 h) [50].The maximal elimination rate (Vmax) of phenytoin is higher for children than adults and an inverse age relationship exists [51].

In general, drugs predominantly metabolized by CYP2C9 may need to be 50-100% higher than those given to adults to achieve equivalent therapeutic concentrations in children [16].

CYP2C19 protein and catalytic activities that were 12-15% of mature values were observed as early as eight weeks of gestation and protein expression increased linearly over the first five postnatal months. At birth, CYP2C19 activity is approximately 30% of adult activity and reaches adults values from 10 years [4].

The proton pump inhibitors omeprazole, lansoprazole, and pantoprazole are all primarily metabolized by this isoenzyme. Clearance of proton pump inhibitors is reduced in neonates but the clearance in children older than one year is the same as in adults [52,53].

In summary, drugs predominantly metabolized by CYP2C19 do not require weight-corrected doses in children older than one year to achieve equivalent therapeutic concentrations [16].

###### CYP2D

CYP2D6 contributes to the metabolism of numerous classes of drugs, such as tricyclic and nontricyclic antidepressants, beta-blockers, antiarrhythmic drugs, codeine, captopril and ondansetron. A clear increase in the CYP2D6 protein expression was found during the first postnatal week. In infants and children up to five years of age, the level had reached about two-thirds of the average adult levels [4].

Pharmacokinetics are similar between children and adults for fluoxetine, paroxetine and risperidone [54,55]. Weight-corrected doses of drugs predominantly metabolized by CYP2D6 in neonates must be decreased. However, in infants, children, and adolescents, weight-corrected doses are approximately the same as those given to adults to achieve equivalent therapeutic concentrations [16].

###### CYP2E

The CYP2E1 isozyme is involved in the metabolism of small molecules, including ethanol or paracetamol. There is controversy whether it is present in fetal liver or not, but it steadily rises after birth, represents 40% of adult values through the first year of life and reaches adult values at 1-10 years [56].

###### CYP3A

Is the most abundant cytochrome in the human liver and the intestinal tract, which accounts for approximately 30-40% of total hepatic cytochrome [16].

Probably essential for the metabolism of steroid hormones of maternal, placental or fetal adrenal origin [4] and for metabolizing more than 50% of the drugs, including ciclosporin, tracrolimus, cisapride, midazolam, fentanyl, lidocaine, nifedipine, indinavir, verapamil [16,43]

CYP3A levels develop at an early stage. CYP3A4 is the major CYP expressed in adult liver, whereas CYP3A7 is the major CYP expressed in the fetal liver. CYP3A5 is more commonly expressed in children and teens and it decreases to 20-30% in adults [56]. Although CYP3A4, -3A5 and -3A7 are structurally closely related, they differ in their catabolic capacity. CYP3A7 is very active in the fetal liver but it progressively declines and reaches a very low level in adult liver.

The activity of CYP3A4 is extremely weak or absent in the fetus and begins to rise after birth to reach 30-40% of the adult activity after one month [57].

The biotransformation of cisapride is mediated by CYP3A4, with a minor contribution of CYP3A7. This explains the lower clearance and higher cardiac toxicity in neonates than in children or adults [56].

Similarly, the clearance of intravenous midazolam, a CYP3A4 substrate, is markedly lower in neonates than that in infants aged >3 months. Furthermore, its bioavailability following oral administration has been reported to be increased in preterm infants compared with that of adults as a result of low CYP3A activity in the intestine [58].

Table 2 shows different half-lives of drugs metabolized by CYP450 isoenzymes between neonates, infants, children and adults.

##### 2.3.1.2. Monoamine oxidases (MAOs)

Monoamine oxidases (MAOs) are oxidative enzymes that are located in the mitochondria and are involved in the metabolism of endogenous as well as exogenous compounds. They are located in a variety of tissues including the liver, kidney, lung, gut, platelets and brain. It has also been described that the MAO A activity in the frontal cortex of human brain was very high at birth, decreased rapidly during the first two years of life and remained constant thereafter [59]. By contrast, MAO B activity was low at birth, remained unchanged during early childhood and increased during advanced age.

#### 2.3.2. Phase II reactions

##### Methylation

The transfer of methyl groups is one of the most extensive reactions in nature. This transfer is carried out by *S*-, *N*-, *O or* catechol-*O*-methyltransferases.

*N*-methyltransferases: *N*7-methylation of theophylline in the newborn to produce caffeine is well developed, whereas oxidative demethylation is deficient and develops over the ensuing months [60].

##### Acetylation

Many drugs such as hydralazine, *para*-aminosalicylic acid, isoniazid, procainamide as well as toxic agents are conjugated with an acetyl group by *N*-acetyltransferase (NAT) [4]. NAT is a cytosolic enzyme widely distributed in mammalian tissues. Its activity in human adult liver cytosol, measured with *para*-aminobenzoic acid, is about three-fold higher than that present in the fetal liver cytosol [61]. Activity of NAT2 is genetically determined and there are two phenotypes: fast-acetylator and slow-acetylator phenotypes. At birth, NAT2 activity is independent of genotype, and the slow-acetylator phenotype predominates. Within the first four years of life, fast-acetylator phenotype in heterozygous and homozygous wild-type individuals develops [62].

##### Glucuronidation

Serotonin (5-HT) is the only substrate to be glucuronidated to the same extent by fetal and adult liver microsomes. In the fetal liver, activities toward bilirubin, androsterone, testosterone and morphine were at values of <14% of those of adults. Bilirubin glucuronidation, carried out by the uridine 5 diphosphate glucuronosyltransferase (UGT) in the fetal liver is <1% of the activity of the adult liver [4]. According to the drug or endogenous compound, glucuronidation may not approach adult values until three months to three years or even later [42]. As in the case of CYPs, the system of glucuronosyltransferases contains a large number of isozymes.

Clearance of zidovudine and lorazepam is decreased in neonates. Oral clearance of ketoprofen, zidovudine, lorazepam and lamotrigine is similar in children and adults [16].

Morphine is extensively metabolized by glucuroconjugation with formation of both 3- and 6-glucuronides (M3G and M6G). M6G has been shown to be a potent analgesic. The metabolism of morphine was studied in children and premature neonates, finding detectable concentrations of M3G and M6G.

The M3G/morphine and M6G/morphine ratios were significantly higher in children than in neonates, suggesting that morphine glucuronidation capacity is enhanced after the neonatal period [63].

Therefore, weight-corrected doses (mg/Kg) of drugs predominantly metabolized by glucuronidation must be decreased in neonates. However, to achieve equivalent therapeutic concentrations, doses for children are approximately the same as required for adults [16].

Table 3 shows phase I and II isoenzyme activities in pediatric population compared to adults and some examples.

#### 2.3.3. First-pass metabolism

Developmental differences in the activity of intestinal and liver drug-metabolizing enzymes that can markedly alter the bioavailability of drugs are incompletely characterized [34]. The intestinal activity of cytochrome P-450 1A1 (CYP1A1) appears to increase with age. In preterm infants, an increased bioavailability of midazolam as a result of a low cytochrome P450 3A activity has been reported in the intestine [58].

First-pass metabolism of zidovudine was decrease in the first 14 days of life. The bioavailability of oral zidovudine varied from 89% in infants younger than 15 days to a mean of 61% in older infants [64,65].

### 2.4. Excretion

Excretion of drugs by the kidneys is dependent on three processes, glomerular filtration (GFR), tubular secretion and reabsorption. They are dependent on renal blood and renal plasma flow, which increase with age as a result of an increase in cardiac output and a reduction in peripheral vascular resistance.

At birth, renal blood flow is only 5 to 6% of cardiac output, 15 to 25% by one year of age and reaches adult values after two years of age [66].

Although during the neonatal period the elimination of many drugs that are excreted in urine in unchanged form is restricted by the immaturity of glomerular filtration and renal tubular secretion, a similar or greater rate of elimination from plasma than in adults has been observed in late infancy and/or in childhood for many drugs including as digoxin, phenytoin, carbamazepine, levetiracetam, diazoxide, clindamycin, cimetidine, chlorpheniramine and cetirizine. [67-70]. Therefore, larger doses of these drugs (mg/kg) are required in children in order to achieve the same plasma concentrations as in adults. The explanations for the lower plasma concentration–dose ratios in infants and children are variable and can be due to a number of phenomena either related to the kidney function (such as an increased capacity of tubular secretion) or not directly related to the kidney function (such as lower plasma protein binding, more extensive tissue binding, increased hepatic metabolic activity, *etc.*).

Finally, infant urinary pH values are generally lower than adult values [66]. Urinary pH may influence the reabsorption of weak organic acids and bases, and differences in renal drug elimination may reflect the discrepancy in urinary pH values.

#### 2.4.1. Glomerular filtration

Inulin or creatinine are often used as a marker of GFR, the former has lower concentration at birth, increases considerably during the first two weeks of life (Table 4) and reach adult levels by six months [66]. However every marker has its own limitations, for instance, during the first days of life, some of the creatinine in plasma may come from the mother and the creatinine clearance may not accurately correspond to GFR [71].

At birth, a direct proportionality exists between gestational age and GFR in the full term infant [72,73]. Furthermore, the increase in GFR correlates with postconceptual age rather than postnatal age; consequently, premature infants on average exhibit much lower GFR values than full term infants. Preterm neonates (33-34 weeks postmenstrual age (PMA)) have a slower increase (13.9 mL/min per 1.73 m^2^ per week PMA) in GFR during their first weeks of life than full term neonates (39-41 weeks, 94.1 mL/min per 1.73 m^2^ per week PMA) [71]. After the first week of life, enhancement in GFR proceeds at the same rate in preterm and full term infants, but even by five weeks of age the absolute value for GFR remains lower in preterm infants [74].

For drugs whose renal clearance is governed by GFR, the rapid improvement in efficiency of glomerular filtration leads to rapid enhancement in renal drug clearance and a diminished risk of significant drug accumulation, such as aminoglycosides [75].

#### 2.4.2. Tubular secretion

The renal tubular secretion capacity increases over the first months of life to reach the adult level at approximately seven months [4]. Therefore, active tubular secretion takes somewhat longer to reach adult values than glomerular filtration. The tubular secretion may be greater in children and teens than in adults.

Functionally, the kidney exhibits a reduced capacity to excrete weak organic acids such as penicillins, sulfonamides or cephalosporins. The renal tubular secretion of high doses of *p*-aminohippurate (PAH), a substrate for the predominant organic anion transport system of the kidney, was only 20 to 30% of adult values at birth and approached adult levels only by seven to eight months of age [66].

These results are consistent with the *in vivo* elimination of furosemide, a PAH transport pathway substrate, where half-lives of 19.9 and 7.7 hours in premature and full term infants, respectively [76], were observed as compared with 0.5 hours in the adult [77].

When renal tubular mechanisms are important in the elimination of a drug, the disproportional rate of development of glomerular filtration and tubular function may have variable and complex effects on the renal clearance of that drug. For example, infant renal clearance values for a given drug may exceed adult values, since a low GFR may be matched by a greater reduction in tubular reabsorption capacity. This has been observed in children of 3-12 years receiving imipenem–cilastatin [78]. In the case of digoxin, the renal tubular secretion also plays a more important role in the excretion of the drug in children and in adolescents than it does in adults [79]; thus, the inhibition of renal tubular secretion by compounds such as amiodarone may cause a steeper increase in serum digoxin concentration in children [80].

#### 2.4.3. Tubular reabsorption

Tubular reabsorption is generally a passive phenomenon especially important with non-metabolized liposoluble drugs. The concentrations of the retinol-binding protein and microalbumin in urine have been measured as a marker of renal tubular and glomerular development and maturation, respectively [81]. The results suggest that the glomerular permeability and the tubular reabsorption are gradual and continuous processes from birth to adolescence, but the key stage of their maturation may be between one and three years, respectively.

## 3. Conclusions

Pharmacokinetic processes: absorption, distribution, metabolism and excretion in the pediatric population are different, to a greater or lesser extent, than those of adults. The deviation even depends on the child's age, making a classification as follows necessary: neonate, infant, child and adolescent.

In general, the absorption, plasma protein binding, metabolism and excretion levels of children are reduced whereas the volume of distribution is increased. However, this is not always certain, as these processes are also dependent on the drug characteristics. Furthermore, it is frequently assumed that the same plasma concentration of a drug and/or its metabolites in pediatric population and adults is responsible for the same pharmacological effect. This statement is far from correct due to the existence of active metabolites, diverse receptor characteristics (quantity and affinity) and variable concentration reached at the site of action, as well as different membrane permeation and plasma protein binding.

All these factors might explain the pharmacodynamic differences between children and adults, and must be taken into consideration when determining the dose (mg/kg) for children, as a function of age, and drug characteristic doses may be greater, lesser or similar to the dose in adults.

Pediatrics studies entail difficulties and ethic limitations, however, they are necessary to determine the posologic regimen of drugs and estimate their fate once administered. Children cannot be considered as miniature adults and extrapolation from adults' data should not be done, especially in long-term treatments.

## Figures and Tables

**Table 1.** Pediatric indication for some drugs by the FDA.

**Drugs**	**Approved pediatric use**	**Drugs**	**Approved pediatric use**	**Drugs**	**Approved pediatric use**
Amikacin	**✓**	Furosemide	**✓**	Pantoprazole	**X**
Amiodarone	**X**	Gabapentin	aged ≥ 3 years	Paroxetine	**X**
Amoxycillin	**✓**	Gentamicin	**✓**	Phenobarbital	**✓**
Ampicillin	**✓**	Hydralazine	**X**	Phenytoin	**✓**
Amphotericin B	**✓**	Isoniazid	**✓**	Propofol	aged ≥ 3 years
Carbamazepine	**✓**	Ketoprofen	**X**	Propranolol	**X**
Cetirizine	aged ≥ 6 months	Lamotrigine	aged ≥ 2 years	Rifampin	**✓**
Cyclosporine	**✓**	Lansoprazole	aged ≥ 1year	Risperidone	aged ≥ 10 years
Cimetidine	aged ≥ 16 years	Lidocaine	**✓**	Sufentanyl	aged ≥ 2 years
Clindamycin	**✓**	Lorazepam	**X**	Teophylline	**✓**
Diazepam	aged ≥ 6 months	Metoclopramide	**✓**	Tolbutamide	**X**
Diazoxide	**✓**	Midazolam	**✓**	Tacrolimus	aged ≥ 16 years
Digoxin	**✓**	Morphine	aged ≥ 1 month	Tramadol	aged ≥ 16 years
Erythromycin	**✓**	Nifedipine	**X**	Verapamil	**X**
Fentanyl	aged ≥ 2 years	Omeprazole	aged ≥ 1year	Warfarin	**X**
Fluoxetine	aged ≥ 7 years	Ondansetron	aged ≥ 4 years	Zidovudine	**✓**

✓ approved for pediatric use from birth

X not approved for pediatric use.

**Table 2.** Different half-lives (hours) between neonates, infants, children and adults.

**Isoenzyme**	**Drug**	**Neonate**	**Infant**	**Children**	**Adult**
CYP1A2	Caffeine	95	7	3	4
	Theophylline	24-36			3-9
CYP 2C9	Phenytoin	30-60	2-7	2-20	20-30
CYP2C19	Phenobarbital	70-500	20-70	20-80	60-160
	Diazepam	22-46	10-12	15-21	24-48
CYP3A	Carbamazepine	8-28	–	14-19	16-36
	Lidocaine	2,9-3,3	–	1-5	1-2,2

**Table 3.** Isoenzyme activity in pediatric population compared to adults and examples.

**Isoenzyme**	**Pediatric population activity**	**Drug class**	**Examples**
CYP1A2	↓ until 2 years	Antidepressant Bronchodilator Diuretic	Duloxetine Theophylline Triamterene
CYP2C9	↓ until 1-2 years	Anticoagulant Antidepressant Nonesteroidal antiinflammatory	Warfarin Phenytoin Diclofenac, ibuprofen, naproxen, tolbutamide
CYP2C19	↓ until 10 years	Antidepressant Benzodiazepine Proton pump inhibitor	Citalopram, sertraline Diazepam Lansoprazole, omeprazole, pantoprazole
CYP2D6	↓ until 12 years	Analgesic Antidepressant Antihistamine Antipsychoticβ-Blocker	Codeine, tramadol amitriptyline, desipramine, doxepin, imipramine, fluoxetine, nortriptyline, paroxetine, venlafaxine Diphenhydramine Risperidone Labetalol, metoprolol
CYP3A4	↓ until 2 years	Analgesic Antiepileptic Antifungal Antihistamine Antiretroviral Benzodiazepine	Alfentanil, fentanyl Carbamazepine Itraconazole, ketoconazole loratadine Indinavir, lopinavir ritonavir, saquinavir Alprazolam, midazolam
MAO A	↑ until 2 years		
MAO B	≈		
N-Methyltransferases UGTs	≈		
	↓ until 7-10 years	Analgesic	Morphine
		Antiepileptic Benzodiazepine	Lamotrigine Clonazepam, lorazepam
NAT2	↓ until 1-4 years	Antihypertensive Antiinfectious	Hydralazine Isoniazid

**Table 4.** Age-related creatinine clearance.

**Age**	**Creatinine clearance** (**mL/min/m^2^**)
Preterms	5-10
1-2 weeks preterms	10-12
Neonates	10-15
1-2 weeks of age	20-30
6 months	73
Adults	73
